# Draft Genome Sequences of Pantoea agglomerans Strains BD1274 and BD1212, Isolated from Onion Seeds, Reveal Major Differences in Pathogenicity and Functional Genes

**DOI:** 10.1128/MRA.01507-19

**Published:** 2020-11-05

**Authors:** Valery M. Moloto, Teresa Goszczynska, Ahmed Idris Hassen, Rian Pierneef, Teresa Coutinho

**Affiliations:** a Agricultural Research Council, Plant Health and Protection, Pretoria, South Africa; b Agricultural Research Council, Biotechnology Platform, Pretoria, South Africa; c Forestry and Agricultural Biotechnology Institute, University of Pretoria, Pretoria, South Africa; University of Maryland School of Medicine

## Abstract

Pantoea agglomerans strains BD1274 and BD1212 were isolated from *Allium cepa* seeds. Strain BD1274 induced a disease symptom on a healthy onion, whereas strain BD1212 did not and remains nonpathogenic. A comparative genomic study revealed that the strains differ in their genomic compositions, particularly in the genes that confer pathogenicity.

## ANNOUNCEMENT

Pantoea agglomerans is a Gram-negative, ubiquitous bacterium that is commonly isolated from seeds, fruits, and plant surfaces and was reported as a human pathogen (1). The bacterium usually occurs as an epiphytic or endophytic symbiont in plants. It has also been identified as a cause of plant diseases, including center rot and leaf and seed stalk necrosis of onion, black spot necrosis of pea, and leaf blight in maize and sorghum, to mention a few (2).

The P. agglomerans strains in this study were isolated from onion seeds by serially diluting each sample and plating it onto tryptone glucose extract agar (TGA; Difco) plates. The plates were incubated at 25°C for 5 to 7 days, after which morphologically different pure colony types were subcultured on TGA plates and incubated at 28°C for 72 h. Further characterization using sequence analysis of the 16S rRNA genes indicated that both strains belong to P. agglomerans with 99.9% nucleotide sequence similarity. In the subsequent pathogenicity trial we conducted, the two strains of P. agglomerans were found to differ in their pathogenicities against onion. P. agglomerans strain BD1274 induced water-soaked lesions on onion seedlings, which turned necrotic after 6 days, while strain BD1212 did not cause any symptoms (3). The genomes of the two strains were sequenced to identify genomic differences between them and to understand possible genetic factors crucial for the emergence of pathogenicity in onions.

For genomic DNA extraction, the bacterial strains were streaked onto TGA (Difco) plates, and the plates were incubated at 28°C for 24 h. DNA was extracted from pure colonies using the Promega purification kit according to the manufacturer’s instructions (Promega, Madison, WI, USA). The genomes of the two strains were sequenced by paired-end Illumina sequencing using the HiSeq 2500 platform (Illumina) at Inqaba Biotechnology (South Africa). Libraries with an insert size of 500 bp were generated for each strain, and sequence lengths of 90 bp in both directions were obtained. The paired-end reads were assembled into contigs using SPAdes v3.9.0 (http://bioinf.spbau.ru/en/spades), and the read quality was determined using Trimmomatic v.0.39 (4).

Annotation was performed using the RAST (Rapid Annotations using Subsystems Technology) v2.0 server, as well as the PATRIC v3.6.3 annotation pipeline (5, 6). Default parameters were used for all software unless otherwise noted. Genomic analysis revealed that P. agglomerans strain BD1274, which is pathogenic on onion, has a larger genome (4,968,508 bp) than does the nonpathogenic strain BD1212 (4,875,404 bp), confirming prior observations that nonpathogens have reduced genomes, compared to those of pathogenic strains of some bacteria (7, 8). BLASTN (https://blast.ncbi.nlm.nih.gov) analysis indicated that the BD1212 and BD1274 genomes each had three plasmids with sequence similarities of 98%, compared with plasmids in P. agglomerans C410P1 (GenBank accession numbers CP016890.1, CP016891.1, and CP016892.1). Additionally, the pathogenic strain BD1274 had a fourth plasmid, similar to that in P. agglomerans strain FDAARGOS-160 (GenBank accession number CP014126). The fourth plasmid in the pathogenic strain contains a cluster of genes that are responsible for the conjugal transfer of DNA, playing a major role in pathogenicity. Major comparisons of the genomic characteristics, including those related to pathogenicity, are presented in Table 1.

**TABLE 1:** Selected major differences in the genomic features of P. agglomerans strains BD1274 and BD1212, based on the PATRIC annotation pipeline

Feature	Data for P. agglomerans strain^a^:	
	BD1274 (pathogenic)	BD1212 (nonpathogenic)
Genome size (bp)	4,968,508	4,875,404
Coverage (×)	20.3	27.0
Read length (bp)	90	90
No. of reads	516,144	710,680
No. of contigs	246	103
DNA G+C content (%)	55	55.1
Total no. of genes	4,918	4,751
No. of protein-coding genes	4,568	4,492
*N*_50_ (bp)	32,581	125,314
*L*_50_	46	13
No. of rRNAs	11	4
No. of tRNAs	53	52
Insertion sequence (transposase-related protein[s])	IS*150* (InsO)	IS*66* (ISS_odB_, InsA)
Cell wall-degrading enzyme	Oligogalacturonate specific porin	—
Type III secretion system(s)	—	HrpB, HrpD, HrpF, HrpJ, HrcQ, HrcB, type III export apparatus
Type IV secretion system(s)	Conjugative transfer of plasmid DNA (TraN, TraG, TraD), transfer of pilus assembly (TraB, TraF, TraC, TraH, TraN), surface exclusion protein (TraT), DNA nicking and unwinding (TraI)	—
Toxin-antitoxin(s)	mRNA interferase (HicA), phage DNA-binding protein (HicB), YefM protein (antitoxin to YoeB), EF hand domain protein	—

a Only genes that are present in one of the strains and absent in the other (—) are indicated to highlight differences.

### Data availability.

The whole-genome shotgun sequences for P. agglomerans strains BD1274 and BD1212 have been deposited in DDBJ/EMBL/GenBank with accession numbers QQXI00000000 and QQXH00000000, respectively; the versions described in this paper are QQXI01000000 and QQXH01000000. The respective SRA accession numbers for the raw data are SRX6718209 and SRX6718208, and both are registered under BioProject number PRJNA481880.
